# Pain in the Foot

**Published:** 1882-04

**Authors:** 


### PAIN IN THE FOOT—CONDITIONS OF ITS OCCURRENCE.

Dr. T. B. Curtis (*Boston Med. Jour.*), gives the following:

1. As an irradiate, reflected, or so-called "sympathetic" pain, in cases of *urethral stricture*, it has been observed by Luxmoor, Brodie, and many others.

2. In cases of *vesical calculus*, "one of the most interesting examples of this," says Von Pitha, "occurred in the person of a colleague, Dr. Reisich, of Prague, who, in 1857, was freed from a large stone by lithotripsy. . . . He indicated, as one of the most precise symptoms of the calculus, a sensation as if he were standing with his left foot on a red-hot plate. With each successive diminution of the size of the stone by means of the operation, the circumference of this burning plate seemed to diminish, and at last only a small edge of the sole remained the seat of this reflected pain. This remnant, however, continued obstinate after I had, as I believed, removed all the detritus, and when exploration failed to detect anything within the bladder. In spite of my denial, the patient insisted determinedly that, according to his sensations, some small fragments still existed at the left side of the fundus of the bladder. I examined him again, and at the end of a few weeks found, at the spot which he had indicated, a small fragment concealed in a fold of the mucous membrane. When this was removed, all pain in the sole disappeared, and the patient, for the first time, expressed himself relieved."

3. Similar irradiate or reflected pains in the foot are occasionally experienced in *cysto-prostatitis* or inflammation of the neck of the bladder. A patient of mine, somewhat advanced in years, had suffered for several years from obstructive hypertrophy of the prostate, with complete retention of urine, requiring the habitual daily use of the catheter, and complicated by chronic catarrh of the bladder. He had also had a phosphatic vesical calculus, which was removed by lithotrity, partly by Professor Guyon in Paris, and partly by me after the return of the patient to this country. He had for several months been afflicted with a most harassing complication, namely, a chronic cysto-prostatitis, liable to frequent acute or subacute exacerbations. A considerable degree of relief, by the way, of the distressing and almost ceaseless vesical tenesmus caused by this inflammatory affection accrued from the use of intraprostatic injections of strong solutions of nitrate of silver—varying in strength from one in fifty to one in twenty parts of water—practised with Guyon's injector, at intervals varying from three or four to six or seven days. In this patient, curiously enough, every paroxysm of prostatic pain, whether caused by movements in bed, by locomotion, by calls to urinate, by catheterism or by the application of nitrate of silver, was and still is liable to be accompanied by a sharp, distinctly localized pain in the ball of the left foot, the sensation then felt being compared by him to that which would be caused by treading with the bare foot upon tacks or broken glass. The pain at the neck of the bladder was of the same description, as if due to the presence of a sharp, jagged foreign body, so as to lead to repeated explorations for calculus under ether ; this pain, also, is left-sided, being referred to the left side of the vesical neck and rectum.

4. In *cystalgia* or neuralgia of the neck of the bladder, Von Pitha himself, suffering from this neurosis, was actually sounded for stone as often as five times, by his own desire, so closely were the symptoms of vesical calculus simulated by his cystalgic paroxysms. A very severe neuralgia at the time also affected the heel, the pain, at its moments of greatest severity, taking on exactly the sensation that would have been experienced if the periosteum were being forcibly separated from the *os calcis*. "The pains are so intense," says Von Pitha, "and the description so complete, that I am under the conviction that if the subperiosteal excision of the *os calcis* were being executed, the pains would only resemble in nature and severity those which I suffer during the acute paroxysm."

5. In *gout*, besides the pain attending the acute inflammatory attacks of dermatitis, lymphangitis, and cellulitis, caused by the deposits of biurate of sodium, sharp pains in the feet, particularly in the heel, at times occur without any objective symptoms Sir James Paget cites pain in the heel as one of the lesser signs of gout. "Of course," he says, "it was not meant that no further inquiries should be made, but only that pain at the heel ought to suggest gout, and lead to further questions on the point. Similarly, pain in the tendo Achillis, especially in elderly persons who had sustained no injury, was due, with very few exceptions, to some degree of gout." Elsewhere he says : "I cannot remember to have heard any patient complain of spontaneous pain in his tendo Achillis, except such as I knew to be by inheritance disposed to gout or a lithic acid diathesis. Pain in the heel of an elderly person has, generally, the same meaning. 'Burning soles' and the less frequent 'burning palms' generally signify a gouty constitution or one closely allied to it, and so do the sensations of hot, tingling, and burning patches of the skin of the thighs, without external appearance of redness or eruption." Dr. Dyce Duckworth, of Saint Bartholomew's Hospital, to whose extensive and painstaking observations we owe much valuable information touching the protean signs of the gouty constitution, testifies to the same effect as follows: "Deep-seated pain in the heel has been recognized as of gouty origin. The sensation is compared to the feeling of a foreign body being implanted there, such as a bullet, and it is noteworthy that this is sometimes a symptom of a renal calculus, which may itself be the outcome of a gouty taint. The pain is sometimes distinctly felt in the tendo Achillis." Charcot mentions the tendo Achillis as one of the ligamentous structures in which uric acid is liable to be deposited in overtly gouty subjects. A discussion took place a few years ago, in one of the medical societies of Paris, on *pain in the heel, or talagia, and its treatment*, in which several speakers took part. It was agreed that the pain was often due to a gouty or rheumatic diathesis, and the use of salicylate of soda, in daily doses of one or two drachms, was said by Bucquoy, Panas, and Desprès, to be often efficacious.

The burning soles and palms, alluded to by Sir James Paget, may, perhaps, be considered more or less analogous to the distressing and at times painful affection of the extremities, recently described by Dr. S. W. Mitchell, under the title of "A Rare Vaso-Motor Neurosis." In this chronic and most refractory disease, however, for which the descriptive name of *erythromelalgia* is proposed by Dr. Mitchell, the subjective sensation of heat or scalding is accompanied by a marked temporary flushing or hyperæmia of the affected parts, with redness and warmth of the surface. This affection, therefore, cannot properly be included in the category of purely subjective ailments considered in this paper.

A patient of my own, an elderly lady, coming from a distinctly gouty stock, who had suffered severely somewhat over a year ago from gallstones, with severe hepatic colic and jaundice, and who has lately placed herself, by my advice, under the care of Dr. J. C. White, on account of a persistent and most distressing generalized pruritis, has also suffered, since last October, from what I take to be erythromelalgia, namely, paroxysms of painful burning of the feet, accompanied, according to her report, by flushing of the skin, and occurring mostly at night, in bed.

6. *Renal calculus* occasionally gives rise to a pain irradiated to the heel, as stated by Dr. Dyce Duckworth in the passage quoted above.

7. In *gonorrhœa* pain in the heel is described by Fournier, Panas, and others, as an occasional manifestation, more or less akin to the various forms and localizations of so-called "gonorrhœal rheumatism," which affects the articular and tendinous synovial membranes and the bursæ. It was first described as affecting the heel in this disease by Swediaur.

8. In *syphilis*, also, according to Fournier, the same phenomenon is met with. Here, as in gonorrhœa, the pain is attributed by him to a bursitis.

9. In *locomotor ataxia* the heel may be the first or, for a while, the principal seat of the lancinating or boring pains characteristic of the first stages of that strange disease. A patient whom I had occasion to see a few years ago, and whom I sounded for stone at the request of his medical attendant, was suffering from occasional painful paroxysms of vesical and rectal tenesmus. For several years previously he had experienced sharp, boring pains (the *douleurs térébrantes* of Charcot), which had first attacked the heel. He subsequently developed ataxic symptoms, with staggering gait. Dr. Seguin, describing the symptoms of locomotor ataxia, says that "patients usually give up in despair the attempt to show you every spot in which they have suffered, and they generally indicate as foci of pain, the heel, instep, and thigh." Dr. Buzzard also expressly mentions the heel as frequently the seat of pain in tabes.

10. Lastly, an obscure form of pain in the feet has been very briefly described by Prof. S. D. Gross, under the name of *pododynia*. It shows itself in the form of great tenderness to pressure, and is met with in certain sedentary classes of artisans, particularly in tailors.—*Detroit Lancet.*

